# The mortality in Gaza in July—September 2014: a retrospective chart-review study

**DOI:** 10.1186/s13031-016-0077-6

**Published:** 2016-05-04

**Authors:** Arild Vaktskjold, Mohammad Yaghi, Usama Balawi, Bjørn Iversen, Wendy Venter

**Affiliations:** Department of Public Health, Hedmark University of Applied Sciences, Elverum, Norway; Department of Research, Sykehuset Innlandet Health Trust, Brumunddal, Norway; World Health Organization, occupied Palestinian territory, Gaza City, Gaza Palestine; Studies and Planning Directorate, Gaza Strip, Gaza Palestine; Norwegian Institute of Public Health, Oslo, Norway; World Health Organization, Regional Office for the Eastern Mediterranean, Cairo, Egypt

**Keywords:** Gaza, Mortality, Palestine, War

## Abstract

**Background:**

The majority of Gazans who were killed or injured in the 2014 Israel—Gaza war were civilians, and one-fourth of the population were internally displaced. As the Gaza Strip is a small territory, the whole population was exposed to the war and its effects on the health care system, supplies and infrastructure. Our aim was to assess the overall, sex and age-group mortality in Gaza for the period July—September 2014 that was not caused by war injuries, and the proportion of non-trauma deaths among adults that occurred outside hospital wards. A comparison was made with the mortality for the same period in 2013.

**Method:**

Date, sex, age, cause and place of each death that was not attributed to war-related physical trauma were collected from death notification forms or death records in Gaza hospitals for the period 01 July to 30 September 2014. The same information was extracted from the local death register for all deaths in the same period in 2013.

**Results:**

The mean age at death was 52.4 years in 2014 and 49.7 in 2013, and about 50 % were older than 60 years in both years. The crude non-trauma death rates among adults were 11.6 per 10,000 population in 2014 and 11.3 in 2013, and the age standardised 13.2 and 12.4, respectively. Higher death rates in 2014 were observed among elderly and women. Cardiovascular disease was the most common cause of death among adults of both sexes, and infectious diseases caused less than 10 % in both periods. Three maternal deaths were observed in 2013 and six in 2014 (*p* = 0.17). The proportion of deaths that occurred in a hospital ward was 71.5 % in 2013 and 51.2 % in 2014.

**Conclusions:**

The mortality from communicable diseases was low in Gaza. We did not detect a higher overall background mortality in the 2014 period compared to 2013, but the observed age and sex distribution differed. The proportion of non-trauma deaths among adults that occurred in a hospital ward was markedly lower during the war. The living conditions and health care situation in Gaza point to the need for close monitoring of mortality.

## Background

The 2014 Israel—Gaza war was the third since 2008. Some 2,220 inhabitants of Gaza were killed and more than 10,000 wounded as a direct consequence of this latest armed conflict [1]. A humanitarian emergency was declared. The majority of the casualties were civilians and about one-fourth of the total population in the Gaza Strip were internally displaced [1–3]. Hospitals, health care centres, ambulances, infrastructure and thousands of homes were destroyed or damaged [1, 3–5]. As a consequence of the long lasting blockade and closure of Gaza, 42 % of medicines and 60 % of medical consumables were at zero stock in government health facilities when the war began [2], and health care workers had not received their wages [4]. These shortages continued after the truce.

War and its aftermath cause severe stress on affected populations due to insecurity and direct impact of the conflict on people, property and livelihoods. Illness patterns change and the need for care increases. In general, the burden of disease in war-affected populations depends on the profile of the society. However, chronically ill and elderly people are particularly vulnerable when societal structures break down and services become less available and accessible. Studies in Kosovo, Serbia and Lebanon indicated that an emergency or war situation aggravates prevalent chronic illnesses and causes excess background mortality [6–10]. In developing countries, the major causes of non-combat mortality during emergencies tend to reflect the public health situation in the country [9]. In Palestine, the epidemiological transition from communicable to non-communicable diseases as the main causes of mortality occurred before the turn of the century [11, 12]. Mortality rate is a key indicator of the humanitarian and public health situation in emergency-affected populations. Reports from past emergencies in various countries indicate that mortality tends to be higher for internally displaced people than for refugees [9].

Most studies of background mortality in war-affected populations have been carried out in camps for refugees or internally displaced persons [9], making the findings difficult to extrapolate to the general population. A challenge in measuring the effect of an emergency on non-combat mortality is that baseline information or an appropriate control group must be available [6, 13]. In Palestine, a system of reporting, registering and monitoring of death in Gaza was in place before the war [14]. A notification form should be filed for each death, and in hospitals a death report is filled out for each case. The present study made use of this paper-filed information.

The aim of the study was to assess the overall, sex and age-group mortality in Gaza for the period 01 July to 30 September 2014 that was non-war related (all deaths that were not directly attributed to injuries caused by the war), and the proportions of non-trauma deaths among adults that occurred outside hospital wards. A comparison was made with the mortality for the same period in 2013.

## Methods

### Context

The 2014 war began on 7 July and a ceasefire was reached the 26 August. The later updated number of fatalities of the warfare was 2,220, which included at least 1,492 civilians (whereof 37 % were children and 20 % women) [1, 3]. The means and targeting of the Israeli military operations also resulted in a high ratio killed to wounded (1 to 4 among adults) [1].

As the Gaza Strip is a narrow and densely populated territory, the whole population was exposed to the war. As a result, the proportion of the population displaced during the war was relatively high even in comparison with large complex emergency situations in other countries [15]. The bombing, shelling and artillery fully destroyed one hospital, five primary health care centres, 19 ambulances and thousands of homes. At the height of the conflict, 11 of 31 hospitals were unable to provide services, and 48 of 97 primary health care centres were closed [3]. When the war ended the number of functioning surgical operation rooms had been reduced from 83 to 54 [4].

The Gaza Strip has 1.8 million inhabitants, with an average population density of about 500 per square km. The natural population growth exceeds three per cent per year. Based on official 2013 figures, the median age was 17.6 years and the average life expectancy 71 years for males and 74 years for females. The territory has been subjected to an international siege and Israeli blockade since 2007, severely affecting the local economy and the supply of fuel, electricity, medication and medical equipment [4, 16]. Existing resources were additionally strained by the war and the influx of injuries and acute health needs (WHO 2014). The majority of Gazans live in poverty [17, 18]. Before the war, about two-thirds of the population received food assistance and about forty per cent were unemployed [5]. Additional details about the situation, population, health services and public health in Palestine and Gaza have been described elsewhere [1, 3, 5, 16, 18, 19].

### Data sources and collection

Death notification in Gaza is based on a standard death notification form (DNF), consisting of a paper copy and three carbon copies, that is completed by hand by a licensed physician. The DNF requires the following information: name, address, age, place and date of death, information about who notified the authorities of the death, direct and underlying causes of death, and additional clinical information.

For deaths occurring in Ministry of Health hospitals, the DNF is completed by the attending physician in the ward or emergency room. The physician also completes a separate form with detailed clinical information concerning the patient’s death that is archived in the patient's medical file. In addition, a discharge form containing information about the patient’s condition, treatment and follow-up is completed for all patients discharged from a hospital, dead or alive. In instances where the patient is deceased on arrival to the hospital and is under 60 years of age, a forensic physician is summoned. The forensic physician obtains verbal information from the family, examines the deceased and fills out the DNF. When an unnatural death is suspected, an autopsy may be requested prior to completion of the DNF. All DNFs completed in a hospital requires stamped approval by the hospital administration. One copy of the DNF is retained in the hospital while the three carbon copies are given to the patient’s family.

Non-governmental (NGO) hospitals do usually not receive terminally ill patients. If a death takes place in an NGO hospital, the hospital is required to issue a medical report and transfer the dead to the nearest governmental hospital where the DNF is completed. If the death occurred outside a hospital, the DNF is completed by a physician called to the location, who provides the three carbon copies of the DNF to the family; alternately the deceased may be brought to a Ministry of Health hospital and the DNF is filled out there.

The family is expected to submit two of the DNFs to the District Primary Health Care Directorate and the Ministry of Health without undue delay. One copy is forwarded to the Ministry of Interior, which requires the stamped DNF and the identity card of the deceased to issue a death certificate. A death certificate is needed for legal procedures following a death, including inheritance.

At the end of each month, the Ministry of Health forwards the DNFs to the Palestinian Health Information Centre in Gaza (PHIC) for entry into the Gaza death register. This registration retrieves the relevant personal data by linking the identity number of the deceased to the Ministry of Interior’s civil database. In the registry, each death should be assigned a code to denote the direct and underlying causes of death according to the 10^th^ Revision of the International Statistical Classification of Diseases and Related Health Problems (ICD-10) [20]. Additional details about the DNF and registration process have been published [14].

For our study, data on all deaths during the period 01 July to 30 September 2013 were extracted from the electronic database of the death registry at PHIC. At the time of the study, the death register had not yet been updated for the period of the 2014 war. Information about all deaths not attributed to war-related physical trauma occurring in Gaza during the period July to September 2014 was instead collected from Ministry of Health hospitals and from the Death Notification Office at the Gaza Primary Health Care Directorate. The DNF was the primary source for hospital deaths, but when not available the death report from the deceased’s medical file was the source. For deaths occurring outside hospitals, the source was the submitted DNF. For the deaths in 2014, a three or four character ICD-10 code was assigned to each case by a trained physician in our study group, based on the same guidelines [20]. The data were checked for missing information about age and sex, as well as for inconsistencies between diagnosis and age and sex. When needed, the primary source was consulted for corrections. Deaths in Gaza attributed to war-related physical trauma, and Gazans who died in a large shipwreck in September 2014 (records not available), were not included in the study. For brevity the terms “non-war related” or “background” are used about the included deaths.

### Data analysis

In our analyses each death among adults (20 years or older) was classified into one of the following categories based on the first two characters of the assigned ICD-10 code [20]:

- Certain infectious and parasitic diseases (ICD: A00-B99)
- Neoplasms (C00-C97)
- Endocrine, nutritional and metabolic diseases (E00-E90)
- Diseases of the circulatory system (I00-I99, except I60-I69)
- Cerebrovascular diseases (I60-I69)
- Diseases of the respiratory system (J00-J99)
- Pregnancy, childbirth and the puerperium (O00-O99)
- Injury, poisoning and certain other consequences of external causes (S00-T98) and external causes of mortality (V00-Z99)
- Other defined causes (not included above)
- Unknown; not sufficiently defined (includes cases where cardio-pulmonary arrest was recorded as the cause of death)

Place of death was classified as either *in hospital ward* or *outside ward* based on the recorded or registered information. Deaths upon arrival in the emergency room were classified as outside. The overall and sex-specific age-standardised mortality rates among adults in each time period were calculated using the direct method and the Segi world standard population weights [21]. In addition, the overall and sex-specific rates were adjusted with the WHO-standard to facilitate for comparisons with mortality rates reported elsewhere [22]. We standardised using 5-year age groups for ages 0 to 79 years, and ≥80 years as the oldest group. The age-standardised rate is a summary of the individual age-specific rates using a standard reference population. This gives the mortality that would have been observed if the study population had the age structure of the standard population.

The mid-year population in each of the two years, as projected by the Palestinian Central Bureau of Statistics, was used as the denominator in estimation of the crude mortality rates (CMR), and in the age-group distribution for standardisation. For comparison of non-trauma mortality among adults in the two time periods, we also estimated the weekly crude rates and monthly crude and age-standardised rates, as well as the proportional distributions of cause of death. Adult was defined as 20 years or older, and all rates are presented per 10 000 population. The death category defined above as ICD S00-T98 and V00-Z99 has for brevity been denoted with the term “injuries”, while the term “non-trauma” has been used when this category is not included.

The chi-square test was used for statistical comparisons of cause-, age-group- and sex-specific frequencies of deaths in the two time periods, while comparisons of counts were analysed with the Poisson distribution-event-test (2-tailed).

This study was carried out with approval from the Palestine Ministry of Health, and in compliance with the Helsinki Declaration. However, the interpretations and views expressed in this article are those of the authors solely and do not necessarily represent the views, interpretations, policies, or decisions of the Palestine Ministry of Health or the WHO. According to the guidelines of the Regional Committee for Medical and Health Research Ethics in Norway, the study did not require ethical approval [23]. The data collected for the study did not include information that identified any individual.

## Results

**Fig. 1:**
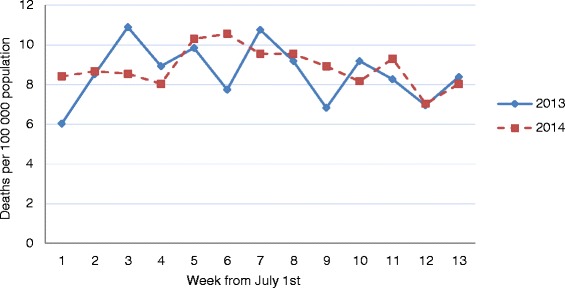
Weekly, crude non-trauma mortality among adults in Gaza in July-September 2013 and 2014

There were 1,241 deaths in July-September 2014 from non-war related causes. The CMR was 7.1 per 10,000 population, compared with 7.2 in the same calendar period in 2013. The sex distribution did not differ for all deaths (*p* = 0.18), or for non-trauma deaths (*p* = 0.21), between the two time periods. Of the 1,241, 718 died during the 51 days of warfare (4.0/10,000), of which 23 % were children. Standardised for age, the rate was 14.6 in 2014 and 14.3 in 2013. The non-trauma CMRs among adults were 11.6 in 2014 and 11.3 in 2013, and the age standardised 13.2 and 12.4, respectively. Higher rates in 2014 were observed for women only. Males accounted for 50.0 % of all deaths in 2014, compared to 52.7 % in 2013 (*p* = 0.18). Additional sex-specific details of the two years are outlined in Table 1. The non-trauma CMR-ratio for adults in August 2014 compared to August 2013 was 1.11 (age standardised ratio: 1.14). In the other two months the CMR was similar between the two years—with September as the lowest in both years. The weekly rates in 2014 peaked in late July to early August (Fig. 1).

**Table 1:** Characteristics of the mortality in Gaza in 2013 and 2014

	July-September 2013^a^			July-September 2014^a^		
	Total	Female	Male	Total	Female	Male
Number of deaths (%)^b^	1232	583 (47.3)	649 (52.7)	1241	621 (50.0)	620 (50.0)
Mean age at death (years)^b^	49.7	53.4	46.4	52.4	55.2	49.7
Crude mortality^b^	7.2	7.0	7.5	7.1	7.2	6.9
Age-standardised mortality^b, c^	14.3	12.7	16.3	14.6	13.6	15.8
Age-standardised mortality^b, d^	17.1	15.3	19.2	17.5	16.3	19.2
Non-trauma deaths (%)	1151	558 (48.5)	593 (51.5)	1198	612 (51.1)	586 (49.0)
Non-trauma death among adults^e^ (%)	859	429 (49.9)	430 (50.1)	926	483 (52.2)	442 (47.8)
Crude, non-trauma mortality among adults^e^	11.3	11.4	11.2	11.6	12.3	11.0
Non-trauma mortality among adults, age-standardised^c, e^	12.4	11.1	14.0	13.2	12.5	14.2
War related adult deaths	1^f^	0	1^f^	1669^g^	299^g^	1370^g^
War related child deaths	0	0	0	551^g^	NA^h^	NA^h^

^a^The mortality per 10 000 population. ^b^Casualties of the war not included. ^c^World (Segi)-standard [21]

^d^WHO world standard. ^e^≥20 years. ^f^OCHA [46]. ^g^OCHA [1]. ^h^Not available

The mean age at death was 52.4 years in 2014 and 49.7 in 2013, and about 50 % were older than 60 years in both years. Compared to 2013, the age-group specific proportions of crude mortality in 2014 was higher in the age-groups above 64 years and lower among infants and in the age group 18–34 (Table 2). Seventy percent of the deaths among adult women occurred in the age-groups older than 64, compared to 61 % among men. As outlined in Table 3, the overall cause-specific distribution of mortality among adults, was different both for women and men in 2014 compared to 2013 (*p* < 0.0001). Cerebrovascular disease was a more common cause among women than men in both years. The largest sex difference between the two years were observed for injuries (*p* = 0.05), cardiovascular disease (CVD) (*p* = 0.24), and the category other causes (*p* = 0.08). In the latter category, renal diseases (ICD-10: N00-N29) constituted about one-half of the deaths in both years.

**Table 2:** Age distribution of deaths and age-group specific mortality in 2013 and 2014 in Gaza

	July-September 2013		July-September 2014^a^		
	Frequency (%)	Crude mortality^b^	Frequency^c^ (%)	Crude mortality^b^	p-value^e^
Infants	221 (17.9)	39.3^d^	194 (15.7)	33.0^d^	0.07
Children 1–4	48 (3.9)	2.1	52 (4.2)	2.2	0.60
Children 5–17	46 (3.7)	0.8	45 (3.6)	0.8	0.96
Adult 18–34	71 (5.8)	1.4	50 (4.0)	1.0	0.01
Adult 35–44	38 (3.1)	2.5	40 (3.2)	2.5	0.79
Adult 45–54	92 (7.5)	9.0	82 (6.6)	7.7	0.32
Adult 55–64	157 (12.7)	28.2	162 (13.1)	27.4	0.71
Adult 65–74	197 (16.0)	74.8	232 (18.7)	84.0	0.02
Adult >74	362 (29.4)	258.6	382 (30.8)	271.1	0.31
Total	1232	7.2	1241	7.0	

^a^Casualties of the war not included. ^b^per 10 000 population. ^c^Age missing in 2 records, whereof 1 adult. ^d^Estimated using 1/5 of the mid-year population in the age group 0–4 as the denominator. ^e^Poisson distribution-event-test of the observed frequency in 2014 (the 2013 frequency used as the expected)

**Table 3:** The sex and cause-specific mortality among adults in Gaza in July-September 2013 and 2014^a^

Cause category (ICD-10)	Overall frequency		Women		Men		p-value
	2013 (%)	2014 (%)	2013 (%)	2014 (%)	2013 (%)	2014 (%)	
Infectious (A00-B99)	73 (8.1)	25 (2.7)	37 (8.3)	12 (2.5)	36 (7.9)	13 (2.8)	0.82^b^
Endocrine (E00-E90)	46 (5.1)	55 (5.8)	25 (5.6)	34 (7.0)	21 (4.6)	21 (4.6)	0.45^b^
Neoplasms (C00-C97)	146 (16.2)	150 (15.9)	72 (16.2)	76 (15.7)	74 (16.2)	74 (16.2)	0.82^b^
Cardiovascular (I00-I59, I70-I99)	230 (25.5)	304 (32.3)	104 (23.4)	153 (31.6)	126 (27.5)	151 (33.0)	0.24^b^
Cerebrovascular (I60-I69)	126 (14.0)	132 (14.0)	79 (17.8)	84 (17.4)	47 (10.3)	48 (10.5)	0.88^b^
Respiratory (J00-J99)	51 (5.6)	48 (5.1)	23 (5.2)	23 (4.8)	28 (6.1)	25 (5.5)	0.78^b^
Maternal (O00-O99)	3 (0.3)	6 (0.6)	3 (0.6)	6 (1.2)	NA^c^	NA^c^	0.17^d^
Injuries (S00-T98, V00-Z99)	44 (4.9)	16 (1.7)	16 (3.6)	1 (0.2)	28 (6.1)	15 (3.3)	0.05^b^
Other	73 (8.1)	95 (10.1)	39 (8.8)	38 (7.9)	34 (7.4)	57 (12.5)	0.08^b^
Unknown	111 (12.3)	110 (11.7)	47 (10.6)	57 (11.8)	64 (14.0)	53 (11.6)	0.16^b^
Total number	903 (100)	941 (100)	445 (100)	484 (100)	458 (100)	457 (100)	0.36^b^

^a^Casualties of the war in 2014 not included. ^b^Chi-square test for independence between sex and years. ^c^Not applicable. ^d^Poisson distribution-event-test of at least 6 deaths (3 deaths as the expected frequency)

The proportion of all deaths among adults classified as unknown or ill-defined cause was 11.7 % in 2013 and 12.3 % in 2014. Of all adult deaths with a known non-war related cause, non-trauma causes constituted 98.1 % in 2014 and 94.4 % in 2013 (*p* < 0.001). Infectious diseases comprised 9.2 % of the known causes in 2013 and 3.0 % in 2014 (*p* < 0.001), and CVD 29.0 % in 2013 and 36.6 % in 2014 (*p* = 0.002). CVD was the largest cause in both years, and caused of 37.4 % of the total known-cause mortality among men in 2014, compared to 35.8 % among women. Three maternal deaths were observed in 2013, and 6 in 2014 (*p* = 0.17). In both years, the cause specific mean age at death was the lowest for maternal deaths, cancer, and in the category *other causes* among both women and men. The largest difference in observed age-group distribution of deaths in 2014 compared to 2013 was for CVD among men (*p* = 0.03), and for respiratory diseases among women (*p* = 0.004). The proportion of adult deaths that occurred in a hospital ward was 71.5 % in 2013 and 51.2 % in 2014 (Table 4). The lowest monthly proportion was observed in July 2014 (43 %).

**Table 4:** Monthly distribution of place of non-trauma deaths among adults (>19 years) in Gaza

	Total		July		August		September	
Place of death	2013	2014	2013	2014	2013	2014	2013	2014
Number of deaths	859	925	293	306	299	345	267	274
Hospital ward (%)	71.5	51.2	70.9	42.9	72.2	52.5	71.3	59.1
Dead at arrival^a^ (%)	7.0	37.5	10.6	43.1	4.9	36.8	5.2	32.1
At home (%)	18.2	10.6	17.1	14.1	18.0	10.7	19.9	6.6
Unknown (%)	3.3	0.6	1.4	0.0	4.9	0.0	3.7	2.2

^a^Includes both cases that arrived the hospital dead and those who died in the emergency room upon arrival

## Discussion

Our study did not detect a higher overall non-war related mortality in July-September 2014 compared to the same months in 2013, but the findings suggested differences in age and sex distribution, and possibly cause distribution, of deaths among adults. The sex ratio shifted towards women and the proportion that was elderly increased, which also occurred in Serbia during the war and the period of sanctions [8].

Extrapolating the overall non-war related mortality estimates for July-September 2014, the annual CMR in Gaza would have been 28.3/10,000, and 58.3/10,000 standardised for age. The difference between the two reflects that the general background burden of death in the Gaza population was low compared to the risk of death because of the young age-distribution of the population. However, in the studied 2014-period the overall death rates would be more than doubled, and among children almost tripled, if we had included people killed by the war. Thus, the death rate was above the emergency threshold indicator of twice the baseline rate [9, 24], and even among women and children the main cause of death in the Gaza Strip during the military conflict was war-related trauma.

The proportion of the mortality attributed to non-communicable diseases was relatively high, partly because of the low mortality from communicable diseases and injuries [12]. Unlike some other post-war situations [9, 25, 26], Gaza avoided widespread violent deaths in the aftermath of the war. The excess mortality reported among internally displaced and conflict-affected populations elsewhere was mainly caused by neonatal disorders and communicable and diarrhoeal diseases [10, 27, 28]. In Gaza, no acute cases of cholera, diphtheria, measles, malaria and tetanus were reported in 2014, and there were no outbreaks of lethal communicable diseases during the war [29], which seems to be another compelling explanation for the relatively low background mortality in Gaza compared to other populations exposed to the atrocities of war. Other explanations might include low mortality rates, high vaccination coverage, and good health status of the population before the war, as well as high resilience, a health care system that was able to maintain or quickly resume most basic operations, and a generous and effective humanitarian response. Of the 25 adult deaths in 2014 classified with infection as the underlying cause, 18 were recorded as unspecified septicaemia and four were acute hepatitis C. In 2013, some 65 of the 73 deaths of infectious diseases were coded as sepsis. Thus, the use of sepsis as the underlying cause partly explains the differences in the observed cause-specific mortality proportion between the two study periods.

The proportional distribution of causes of death in a population is heavily influenced by age structure and birth rate. We expected an increase in frequency of infant deaths in 2014 since the number of births increases year by year, and since findings in a recent study suggest that the previous steady decline in infant mortality in Gaza may have halted [30]. Thus, there might be infant cases missing in our 2014 data. The distribution among adults, on the other hand, is easily comparable between populations. Our data suggested that the CVD-mortality increased in 2014, especially among women and older men, while infectious diseases and injuries decreased as causes. An association between war conditions and increase in CVD-mortality was also observed in Lebanon [6] and Kosovo [31], and the prevalence of heart disease among Bosnian refugees in Croatia was twice that of the local population [32]. Adverse cardiovascular events seem associated with both acute and chronic emotional stress [33], and the direct association between post-traumatic stress disorder and the risk of CVD has appeared stronger among women than among men [34]. Maternal deaths have been closely monitored in Gaza, and the total number in the year 2013 was 12 [2]. In 2008 the figure was 11, and 19 in 2009, and underestimations have been likely to occur [35]. In this perspective, six documented cases in our three-month study period in 2014 is of concern. The fluctuating figures from year to year may reflect random variation, but at the same time the figures suggest that the maternal mortality situation in Gaza is not improving.

The weekly and monthly crude non-trauma death rates revealed a more stable week-to-week rate among adults in 2014 compared to 2013, and lower rates in September for both years. Although September is one day (3 %) shorter than the other two months, the decrease in September suggests a seasonal influence on mortality. According to United Nations Office for the Coordination of Humanitarian Affairs, the emergency situation peaked in the last week of July 2014 [1], and our data indicate that the background mortality among adults reached its highest level during the same week and the week after (Fig. 1). However, there were two weeks in 2013 with crude rates at similar levels. The mortality pattern did not seem influenced by the holy month of Ramadan, which ended on 26 July 2014 and a week later in 2013.

Less than fifty per cent of non-trauma deaths among adults in the first month of the war occurred in a hospital ward, but the proportion gradually increased over the course of the study period. In 2013 this proportion was stable at above seventy per cent from month to month, which was relatively high for a developing country [36]. Our data indicate that a substantially larger proportion in 2014 died on the way to, or in, the emergency room, and that the difference between the two years was pronounced among the elderly. The proportion that died in a hospital during the warfare, and its decrease from pre-war levels, were similar to that observed in Iraq in 2003 [25]. The lower proportion that died in hospital ward in 2014 may also explain the lower use of sepsis as the recorded underlying cause in that period (83 % of all who were coded sepsis in both years died in hospital ward). In addition, review of the available death notification forms in the 2014 data collection facilitated establishment of the underlying cause that resulted in sepsis.

The effects of a complex emergency on death rates for specific non-communicable diseases depend not only on the pre-emergency public health situation, but also on the duration of the crisis and how it affected the functioning of the health services, and the access to them [31]. In the present study we focused on short term impacts of the most recent war on mortality, but the mortality in both 2013 and 2014 were also results of long term and past impacts. The previous intensive military strikes on Gaza took place less than two years earlier, and the population has lived under occupation and conflict condition since long before that time [16]. Reportedly, it takes four to six months before mortality returns to pre-crisis levels after an effective humanitarian response to a crisis [37], but the impact of stress on the risk of death may last longer [33, 34]. A study in Lebanon found that repeated exposure to war events increased the risk of non-combat mortality [6]. Thus, even though we consider 2013 the best available year to use as a baseline reference for mortality comparisons, the 2013 comparison may not have been optimal for detecting the real effects of the war situation on the background mortality. Instead, the level of the absolute mortality rates, both in 2013 and 2014, were reflecting the impact of the very taxing living conditions, the ongoing conflict, and the repeated military escalations on public health in Gaza over time. With data for additional years back in time, we could have taken the underlying trends in mortality patterns into account, which would have strengthened our study. However, initiatives to improve death registration [38], and sub-sequent changes in the DNF in 2012, likely influenced the comparability of data before 2013 with those of 2013.

Detection and reporting of deaths is a challenging issue for any registration system [38], and more so in a complex emergency situation [28]. Studies of mortality in populations exposed to war have usually been based on burial-site surveillance, or sample or cluster surveys [6, 7, 9, 13, 25, 26, 28, 39], either because of lack of a death registration system or because the system of reporting and registration disintegrated [13, 39]. Underreporting or undercounting of deaths, as well as over-estimation of population size, tend to be common in such settings [27], which leads to underestimation of crude mortality. An assessment of death registration in the occupied Palestinian territories in 2004 found 75 % completeness [40]. There has been no time limit for submission of the DNF to the authorities, which may have contributed to undercounting of deaths. However, in our study this possibility pertained only to deaths that took place outside a hospital, as we also collected data from the death reports in the hospitals. Furthermore, a delay beyond our data collection in December 2014, or no submission at all, was likely rare for adult deaths since a death certificate is necessary for inheritance claims, and for the survivors to receive benefits.

Possible explanations for the lower death figure among men and the low mortality from injuries in our 2014 data include war trauma as a competing risk, and that some traumatic fatalities that were not war related were filed as such. However, the number was probably not large, as the number of deaths due to injuries was low also in 2013. On the other hand, it is plausible that less people died in traffic, of drowning, and of work related causes during the war. An under-ascertainment in 2014 of a magnitude equal to the difference in injury deaths between the two years (28 deaths) would only have increased the CMR in 2014 from 7.1 to 7.2 per 10,000 population. The reported non-trauma figures and findings, including Tables 3 and 4, and Fig. 1, would not have been affected.

A typical challenge in studies assessing mortality in a population is the occurrence of deaths elsewhere than within the political boundaries of the population. Because of the lack of certain treatments and specialist services in Gaza, the number of referrals to the West Bank, Egypt and Israel was high before the war, but death notifications were sent to Gaza if patients died while outside the territory of Gaza. The closure of the main border crossing (Rafah) in 2014 affected the possibilities for referral, and the number of regular referrals declined by 32 % in July and 46 % in August 2014 [41, 42]. Nevertheless, of the 474 adults who died in a hospital ward of non-war related causes in July to September 2014, seventeen occurred in a hospital outside Gaza (10 in the West Bank and 7 in Israel).

The limited number of deaths in the studied periods curbed the possibility for more detailed assessment and comparison of causes and age groups. Concerning denominators, the population in Gaza has since 2007 largely been contained within its boundaries because of the physical closure of the territory. During the 2014 war, other than the regular referrals and the 465 casualty patients transported to hospitals outside Gaza (mostly to the West Bank) [42], very few Gazans were permitted to leave the fenced-in enclave. Thus, the accuracy of the available population figures mainly relates to the precision of the sex and age group specific population projections of the Central Bureau of Statistics, which have been based on the 2007 census [43]. However, any inaccuracies in the population and age-distribution projections were presumably not differential between 2013 and 2014.

A notorious shortcoming of vital registration systems and mortality studies based on hospital and official records is misclassification of the underlying cause of death [36, 44], even for deaths that occur in a hospital [36]. Studies in different countries have identified common underlying causes of death that are frequently diagnosed incorrectly, namely ischaemic heart disease, diabetes mellitus, cerebrovascular disease and external causes of injury. Typical errors were to register diabetes mellitus as ischemic heart disease, and ischemic heart disease as another CVD or as diabetes mellitus [36]. Because of the limited study size, we classified the causes only on a 2-character level, thereby also avoiding the misclassifications that are common on a 3-character level. Although misclassification between diabetes mellitus and CVD likely also was present in Gaza [45], the sex- and year-specific tendencies of these two causes were similar. Thus, our findings would not have changed by merging them into one category; instead the tendencies would have strengthened.

We obtained the 2013 data from the death register, while the 2014 data were collected in the hospitals and the cause of death coded separately, which may have influenced comparability of cause-specific figures between the two years. An assessment of Gaza mortality statistics based on 200 randomly selected hospital deaths in 2012 (37 % children) concluded that the primary sources and the DNF more often correctly stated the underlying cause, compared with the coding in the death register (69 % agreement in coding on chapter level), and that the hospital records were more correct than the DNF (74 %). The proportion in agreement was higher for adult deaths than for children. Incorrect coding of cause was most common for metabolic diseases, while cancer had high congruence [45]. The stability of the cancer mortality rates between the two years in our study supports the latter finding, and suggests that the completeness of the death recording was similar in 2014 and 2013. However, there are likely inaccuracies present in the proportional distributions of other cause categories than cancer and maternal mortality in Table 3.

In 2003, the mortality data in Palestine were classified as medium quality in 2003 according to WHO-criteria, as about 20 % of causes were ill-defined [11]. The classification was based on the national database of mortality that had been operational since 1994. In recent years the Ministry of Health has collaborated with the WHO and the Norwegian Institute of Public Health to strengthen the system of registration and reporting [14]. However, the 2014 war delayed the implementation of procedures that describe how to fill out the DNF and the responsibilities of the various actors in the process of registration. Thus, the understanding of the process has varied within the health information system, which likely also affected the quality of the recorded and registered causes of death [38]. Consequently, the revealed increased proportion of deaths that took place outside hospital ward in 2014, in addition to the war situation itself, may have influenced misclassification patterns.

An indicator of quality of death registration is a low proportion of deaths coded as “Symptoms, signs and abnormal clinical and laboratory findings, not elsewhere classified” (ICD-10:R00-R99), cardiac arrest or heart failure [44]. In 2014, the majority with an ill-defined cause was older than 74 years, which was disproportionally high. However, no sex difference was observed, and the relative magnitudes of our estimated cause-specific mortality proportions were biased only if the real underlying causes of the ill-defined cases were not proportionally distributed among the defined cause categories. Information about sex was available in all records, while age was missing in only two, which are important quality indicators [44]. Thus, the total rates, and the sex- and age-specific figures (as presented in Tables 1 and 2, and Fig. 1) were not affected by misclassification or ill-defined underlying cause.

The physician who determined the cause of death could subsequently have forwarded certain cases for forensic examination. Of the 372 deaths in September 2014, eighteen (4.8 %) were forwarded, including ten adults. However, the ascribed cause of death remained the same for all the forwarded cases. Possible explanations for this include a low standard of the forensic laboratory and the fact that most families object to autopsy or incision of the body. The death-registration system does not have insight to the forensic work and elaborations.

## Conclusions

The largest cause of non-war related deaths for adults in Gaza during July-September 2014 was CVD, and the mortality from communicable diseases was low. About 50 % of all who died were older than 60 years of age. We did not detect a higher overall background mortality in Gaza in the 2014 period compared to 2013, but the adult mortality in 2014 moved towards a higher proportion in the oldest age groups and among females. The proportion of non-trauma deaths among adults that occurred in a hospital ward was markedly lower during the war. The living conditions and health care situation for the people in Gaza point to the need for close monitoring of mortality.
